# Indoor Color and Space Humanized Design Based on Emotional Needs

**DOI:** 10.3389/fpsyg.2022.926301

**Published:** 2022-07-13

**Authors:** Yunkai Xu, Shan Wu

**Affiliations:** ^1^School of Textile Apparel and Design, Changshu Institute of Technology, Suzhou, China; ^2^Faculty of Art Design, Guangdong Baiyun University, Guangzhou, China; ^3^Graduate School, Sejong University, Seoul, South Korea

**Keywords:** emotional needs, interior design, design color, space humanization, neural network model interior space design

## Abstract

The increase in emotional consumption reflects the increased emotional appeal of people in modern life. As a place for people’s daily life and consumption, the indoor environment has been regarded as a symbol of quality of life and esthetic taste. The purpose of this paper is to study how to analyze and study the color factor and space humanization in interior design based on emotional needs, and describe the neural network. This paper puts forward the problem of emotional needs, which is based on the neural network model, and then elaborates on its concept and related algorithms, and designs and analyzes the case design and analysis of the humanized design of interior color and space based on emotional needs. The experimental results show that in the evaluation of the emotional needs of indoor environment users, the emotional needs of users for the three levels are all above 3.00. Users have the highest emotional needs at the usage level, reaching 4.24. It shows that users pay more attention to the practical value of the indoor environment, and hope to obtain a pleasant emotional experience by meeting the needs of practical value.

## Introduction

The indoor environment has the dual properties of material and spirit. In modern society, with the increase of work pressure, the alienation of interpersonal relationships, and the weakening of human emotions, people’s emotions need a channel for relaxation, liberation, and satisfaction. People are no longer satisfied that the interior environment only focuses on meeting functional needs but are more concerned about whether the interior environment can meet spiritual and emotional needs.

How to design a better interior decoration space and express people’s needs for indoor use at the current stage is the problem discussed in this paper. Starting from people’s psychological and behavioral needs, this paper analyzes the relationship between people’s emotional needs and interior design. In order to explore the relationship between indoor space and psychological behavior, the color of the design and the humanization of the space have a great impact on the satisfaction of users’ emotional needs. This provides a theoretical reference for interior decoration, summarizes the expression skills and design factors in the feeling, and improves the practical significance of the feeling and design.

The innovation of this paper is: (1) this paper combines emotional needs with artificial neural networks, and introduces the theory and related methods of neural network in detail. (2) In the face of the emotional needs of the occupants, this paper analyzes the degree of emotional needs of the occupants in the indoor environment. Through the evaluation of the experimental results, it is concluded that in the interior environment design, when the designer chooses the color, the first thing to understand is the relationship between the color and the feeling. Reasonable color matching will make people feel positive emotions. Therefore, the first principle of using color in different interior environment designs is to meet the needs of users.

## Related Work

Emotional needs belong to the spiritual level, and it is a process of gaining a sense of identity psychologically. Design is always accompanied by the dual qualities of material and spiritual, and the space combines the dual functions of emotion and material. Uzun and Yldrm (2018) customized a simplified version of the popular numerical reasoning game Sudoku, to study the effects of scaffolding demonstrations plus reward mechanisms on problem-solving behaviors and actions aimed at leveling up. The results show that the reward mechanism promotes independent problem solving rather than relying on scaffolds, and the addition of scaffolds and reward mechanisms encourages experienced players to create new rules, overcome the limitations of existing rules, and develop more complex learning strategies. He discusses the need to carefully design the types of scaffolding presentations for specific instructional purposes, and the potential benefits teachers can have in analyzing the difficulties individual students face in solving numerical problems. However, his design is not innovative enough. Swasty and Adriyanto (2017) aimed to examine the effects of incorporating different emotional design approaches into multimedia on positive emotions, mental engagement, and learning achievement (recall and transfer) among seventh-grade middle school students. The results show that positive emotions generally increase as the number of emotional design features increases. However, his experiments have many factors. Jing and Lv (2021) proposed a convolutional neural network model based on multicolor space and builds a convolutional neural network based on VGGNet (Visual Geometry Group Net) in three different color spaces, namely RGB (Red Green Blue), LAB (Luminosity a b), and HSV (Hue Saturation Value) color spaces. When Okur (2020) touches consumer buying behavior through emotional design, he finds three important attributes: emotion, reasoning, and perception. Then, as he further explains emotional design, he touches on three levels of design: instinctual, behavioral, and reflective. To design something is to design emotion, thereby creating vivid and symbolic images of high artistic value. However, its practical significance is not large. Le et al. (2018) used effort-related physiological measures (i.e., heart rate variability) to examine the impact of emotional design principles on an individual’s investment in mental effort. The findings are consistent with the affective mediation hypothesis of cognitive-affective media learning theory, suggesting the potential importance of including affective and motivational factors in multimedia learning research. However, his content is not detailed enough. Sun (2022) introduces neural network modules such as 3D spatial convolutional (3DSC) neural networks and fuzzy neural networks (FNN), and a deep learning algorithm of indoor spatial layout design (ISLD) based on the adversarial neural network (ANN) is formed. Zeng and Wang (2017) analyzed and summarized the emotional factors of products on the basis of previous research in order to help use the progressive relationship in product design to deepen the four levels of emotional application, and summarized the manifestations of product emotional design. Based on the above analysis, the four design levels involved in the realization of emotional design products and the hierarchical relationship between the four levels are summarized. However, his innovation is not enough. For the relatively special field of industrial robots, Zhang et al. (2019) present a novel learning-based framework for video content-based advertising, DeepLink, which aims at linking Sitcom-stars and online shops with clothing retrieval by using state-of-the-art deep convolutional neural networks (CNNs).

## A Neural Network-Based Approach to Emotional Needs

### Hierarchy of Emotional Needs

#### Emotional Needs

**Figure 1:**
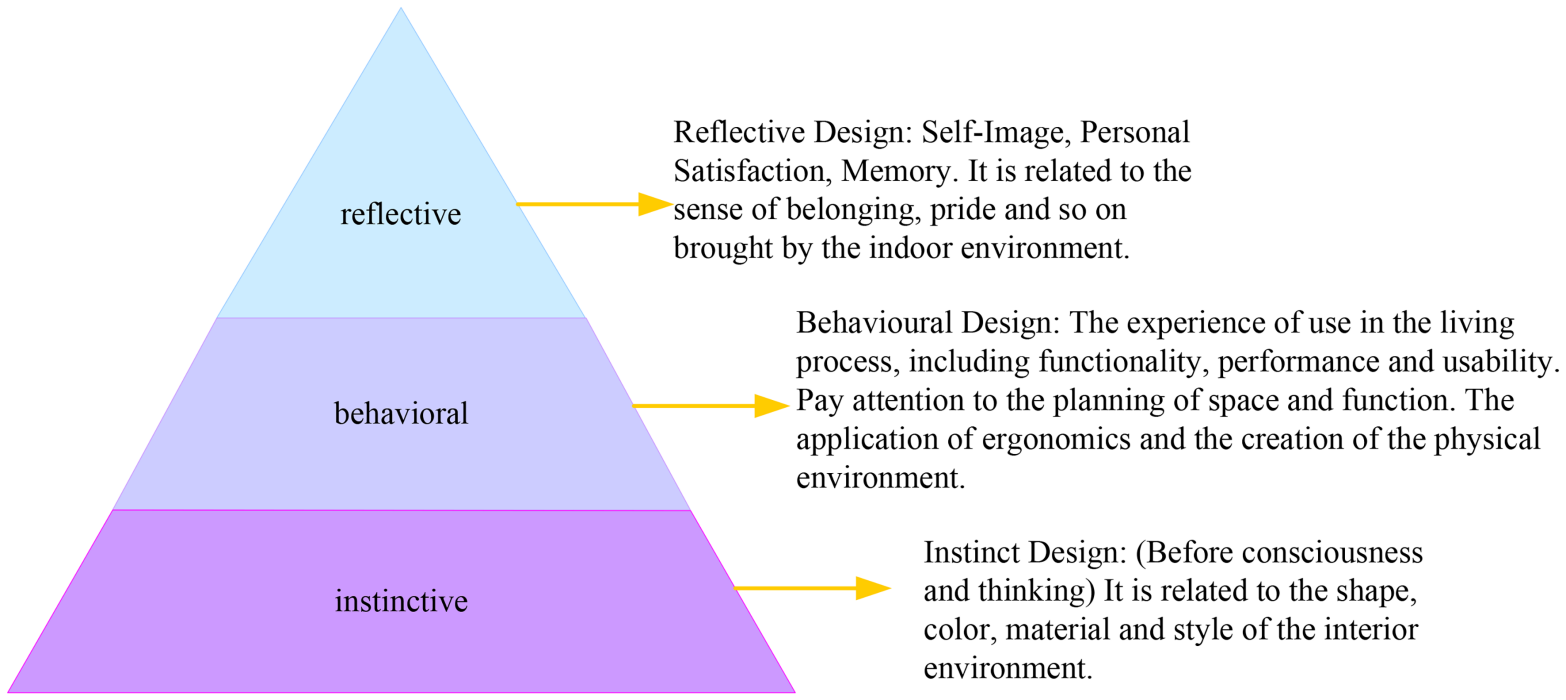
The application of emotional design theory in interior design.

Emotion is a value judgment system of people. It judges the good or bad or safety and danger of things and the environment from the true state of people, and makes people produce corresponding behavioral responses to seek advantages and avoid disadvantages. Both positive and negative emotions are essential to help people survive better. Introducing emotions into contemporary design concepts and grasping and using human emotions in design can create more humanized and popular design works. The application of emotional design theory in interior design is shown in Figure 1.

#### Color Expression

Color is an important part of the visual design of a space, especially for occupants who have a unique association with color. Different colors bring different psychological feelings to the occupants. For example, seeing red is associated with the sun, passion, tradition, and blood. Green is reminiscent of plants, lake water, environmental protection, and health. Different color combinations will also bring different psychological feelings, and different color combinations will also change the atmosphere of the entire room. Relatively speaking, the richer the color, the more complex the spatial level (Cortés-Villalba et al., 2017).

#### Lighting Expression

Light sources are divided into natural light sources and artificial light sources. Artificial lighting is widely used in shopping malls. The function of lighting design is divided into three categories: basic lighting, accent lighting, and decorative lighting. Among them, the basic lighting belongs to the top lighting, the position is high, and the light distribution is relatively uniform, which is convenient for the overall environment of the projection space and meets the general lighting needs. It is the main way of lighting for public and transitional spaces. Accent lighting is very bright and bright enough to grab people’s attention at the same time, mainly for highlighting products. Compared with the first two kinds of decorative lighting, the lighting function is not so obvious; usually, the lamps have a decorative effect and play a role in decorating the indoor atmosphere (Yu and Xiaoting, 2018).

#### Modeling Expression

Formal beauty needs to be reflected in space modeling. Formal beauty is the cognition and formal generalization of beauty formed in long-term production labor and social life. If the esthetic sense of form is highlighted in the space design, it will bring a more perfect visual and psychological experience to the audience. In the creation of formal beauty, the design needs to be combined with the functional layout, and on the premise of satisfying practical functions, explores the psychological suggestion effect of space modeling on people’s emotional experiences and psychological feelings (Stark et al., 2018).

#### Expression of Material

In interior design, the choice of materials not only determines the quality of the design, but also affects the visual and sensory effects of the space. Different materials bring different sensory stimuli to people’s nature, and the texture, color, temperature, etc., of the materials will stimulate people’s different associations. In this era of pursuing fashion and trends, fashionable materials can better cater to women’s psychology.

The impression of metal materials is usually cold, sharp, and extremely reflective, which symbolizes good quality. Different processing technologies make the material have different properties. Glass is one of the more commonly used materials in modern interior design. It can not only separate the space, but also play the role of decorating the space. It is loved by the majority of designers. Glass is generally transparent and has a crystalline texture, which symbolizes purity. With the development of indoor technology, various anti-natural materials have appeared on the market, such as artificial stone, artificial wood, etc., even to the extent of being fake, but these artificial materials have also become the main source of indoor air pollution. And natural materials are still irreplaceable in lives (Francisco and Garcia, 2020).

### Artificial Neural Network

Artificial Neural Networks (ANNs) have the ability to store and use prior knowledge, and also have the ability to analyze emotional needs, such as self-adaptation, self-learning, parallel processing, nonlinearity, fault tolerance, and reasoning capabilities. The good properties make neural networks very suitable for dealing with inaccurate knowledge, such as emotional needs analysis, mainly including causal relationships, where there may be contradictions and errors in the data (Thornquist, 2017; Bao et al., 2021). In view of this, this paper applies artificial neural networks to emotional needs analysis system, connects emotional needs with neural networks, and establishes emotional needs analysis system through sample training.

#### Biological Neural Network Model

No amount of material consumption can bring the emotional pleasure obtained spiritually. Excessive material life will also bring people a sense of emptiness and decadence. Paying attention to the combination of emotion and material and the happiness of life will be gradually satisfied. The indoor environment is the place where people spend the most time living, working, studying, and communicating. The expression of interior design reflects their lifestyle and esthetic concept. On the basis of functional requirements, the abstract emotional concept is integrated into the design to make it a perceptible form of expression to obtain the missing emotional needs. This is a new demand for interior design, and it is also an interior designer Content to be thoughtful about.

**Figure 2:**
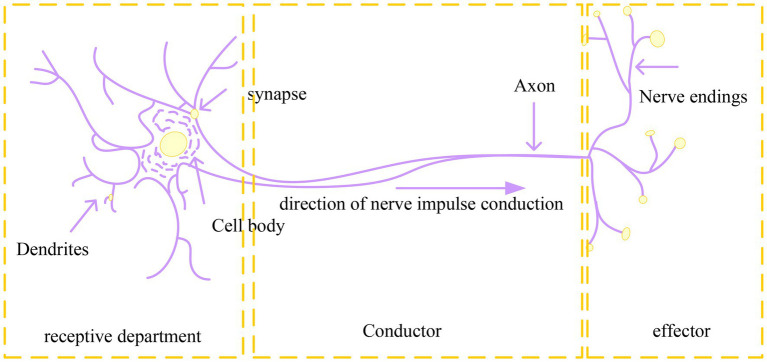
Schematic diagram of neuron composition.

The basic structure of the human nervous system is the neuron (or nerve cell, as shown in Figure 2), which is the most basic unit that transmits information to the human body. According to the research data of neurobiologists, the human brain usually has about 1,010–1,011 neurons (Qu and Guo, 2019). Nerve cells in the human body are composed of three parts: cell body, axon, and neuron. Axes are used to output signals to other neurons, and since axes have many nerve endings, many neurons can receive signals. Dendrites are used to receive signals from other neurons. The cell body of a neuron is the equivalent of a computer’s CPU, which processes the signals it receives and then extracts the signals from the axis. The part of a neuron’s dendrites that connects with the nerve endings of other neuronal axes is called a synapse.

#### Artificial Neuron Model

**Figure 3:**
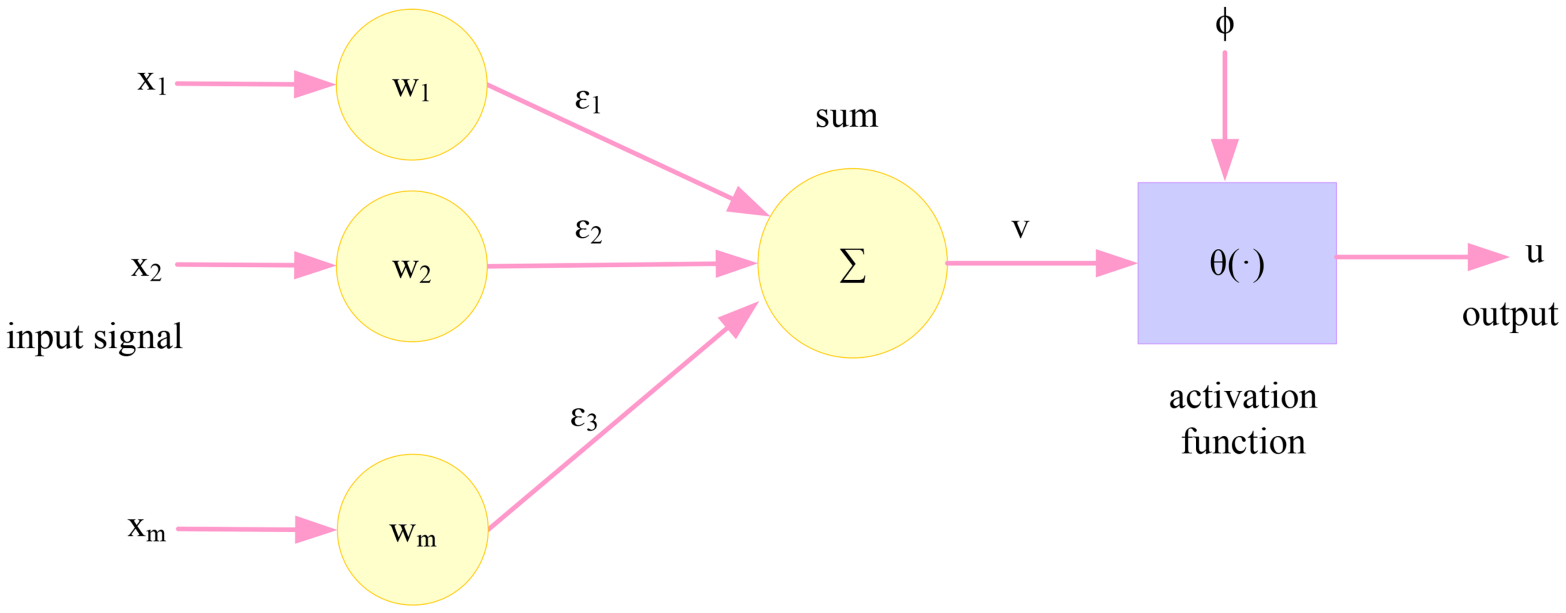
Basic neuron model.

The basic unit of artificial neural network is called artificial neuron, and its model is shown in Figure 3. It is similar to a nonlinear thresholding device with multiple inputs and only one output.

It has a set of connections, and the weight of each connection is represented by a weight. If the weight is greater than zero, the connection is opened, and the connection less than zero is closed.

It has a summation function to obtain the weighted sum of the input signals.

Non-linear activation mode, commonly used activation functions θ(·) are sigmoid function, piecewise linear function, and threshold function, etc., and a threshold *ϕ* (or bias-θ).

Defining the input vector for the neuron:


(1) $$X={\left[X_{1},X_{2},X_{3},\ldots ,X_{m}\right]}^{Z}$$

Defining the weight vector *ɛ*:


(2) $$\varepsilon ={\left[\varepsilon_{1},\varepsilon_{2},\varepsilon_{3},\ldots ,\varepsilon_{m}\right]}^{Z}$$

*ϕ* is the neuron’s threshold and θ(·) is the neuron’s activation function. Then, the u neuron output vector is:


(3) $$u=\left(\overset{M}{\underset{b-1}{\sum }}X_{b}\varepsilon_{b}+\varphi \right)$$

#### Learning of Artificial Neural Network

##### Error Correction Learning (Delta Rule)

Let $y_{t}\left(m\right)$ be the actual output of neuron *t* at time m when x(m) is input, and $d_{t}\left(m\right)$ is the expected output, then the error signal is:


(4) $$e_{t}\left(m\right)=y_{t}\left(m\right)-y_{t}\left(m\right)$$

Error-corrected learning is based on the minimum value of the objective function $e_{t}\left(m\right)$ , so the actual exit of each neuron to the neural network is statistically closer to the exit of the sample.

The most commonly used objective function is the mean squared error criterion, defined as:


(5) $$K=E\left(\frac{1}{2}\underset{t}{\sum }e_{t}^{2}\left(m\right)\right)$$

Among them, *E* is the expectation operator. Because when *K* is used directly as the objective function, it is necessary to measure the properties of the whole process. To solve this problem, we usually replace *K* with the instantaneous value ϕ(m) of *K* at time m, namely:


(6) $$\phi \left(m\right)=\frac{1}{2}\underset{t}{\sum }e_{t}^{2}\left(m\right)$$

Using the steepest gradient descent method, we get:


(7) $$\Delta w_{tk}\left(m\right)=\gamma \left(m\right)e_{t}\left(m\right)x_{k}\left(m\right)$$

Among them, γ(m)>0 is the learning step size.

The neural network model of this learning rule is very extensive, such as the simplest perceptron learning algorithm, which is also the most primitive application of neural network, and the most classic application is the back-propagation learning algorithm, also known as the BP algorithm.

##### Hebb Learning

Neuropsychologists believe that “when neurons on both ends of a synapse (connection) fire in synchrony (and activate or inhibit), the strength of the connection should increase, and vice versa.” It is mathematically expressed as:


(8) $$\Delta w_{tk}\left(m\right)=G\left(y_{t}\left(m\right),x_{k}\left(m\right)\right)$$

Among them, the states of neurons at both ends of $w_{kj}$ are $y_{t}\left(m\right)$ and $x_{t}\left(m\right)$ , and the most common cases are:


(9) $$\Delta w_{tk}\left(m\right)=\gamma y_{t}\left(m\right)x_{k}\left(m\right)$$

Since Δw is correlated with $y_{t}\left(m\right)$ and $x_{t}\left(m\right)$ , it is also called correlation learning rule.

There are two types of networks that apply this rule: discrete Hopfield networks and continuous Hopfield networks.

##### Competitive Learning

In the competitive learning of the network, each output unit competes with each other, and finally only one of the strongest can be activated. The rules are represented by the following formula:


(10) $$\Delta w_{tk}=\{\begin{array}{c} \gamma \left(x_{k}-w_{ki}\right),\mathrm{If neuron}\;k\;\mathrm{competes to}\;\mathrm{win}\\ 0,\;\;\mathrm{If neuron}\;k\;\mathrm{fails to compete} \end{array}$$

When the environment in which the learning system is located is stable (statistical features do not change over time), in theory, the statistical features of the environment can be learned through supervised learning. These statistical features can be used as an experience by the neural network to remember that the environment is non-stationary (Vaidya and Kalita, 2021). Usually, supervised learning does not have the ability to monitor such changes, and to solve this problem, the network needs to have a certain adaptive ability.

#### BP Neural Network

Interior design is to meet the special needs and emotional needs of some people in a targeted manner. Color and space are the core of space design. Different space types satisfy different groups of people with the change of color. At present, the consumer groups targeted by interior design are mainly young people who like romance and sentiment, and the middle class who pay attention to the quality of life. Therefore, when designing emotional needs research, it is necessary to conduct in-depth research and analysis on the psychological behavior of consumer groups. The following is a brief analysis based on the BP neural network.

**Figure 4:**
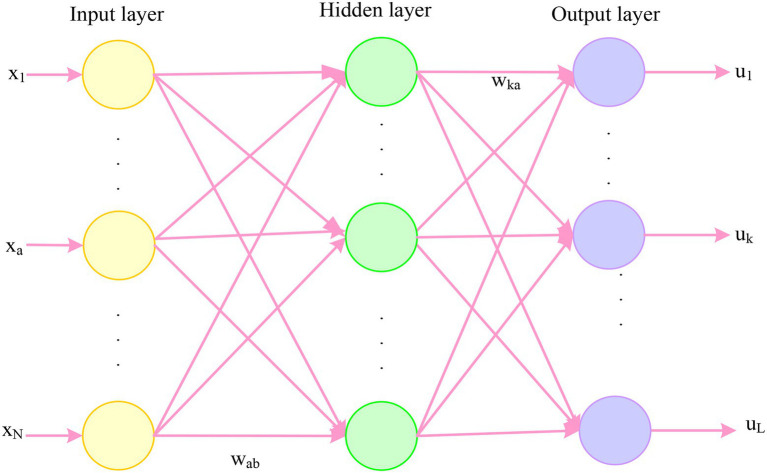
BP neural network structure.

The basic BP network algorithm includes two propagation directions: forward propagation of the input signal and backward propagation of the output error. That is, when calculating the actual network output, it is performed in the direction from input to output, but the weights at each network level are calculated and reversed from output to input, as shown in Figure 4.

##### The Forward Propagation Process of the Input Signal

The input $net_{a}$ of the a-th node of the hidden layer of the network:


(11) $$net_{a}=\overset{N}{\underset{b=1}{\sum }}w_{ab}x_{b}+\varphi_{a}$$

The output $y_{a}$ of the a-th node in the hidden layer of the network:


(12) $$y_{a}=\phi \left(net_{a}\right)=\phi \left(\overset{N}{\underset{b=1}{\sum }}w_{ab}x_{b}+\varphi_{a}\right)$$

The input $net_{k}$ of the kth node of the network output layer:


(13) $$net_{k}=\overset{q}{\underset{a=1}{\sum }}w_{ka}y_{a}+i_{k}=\overset{q}{\underset{a=1}{\sum }}w_{ka}\phi \left(\overset{N}{\underset{b=1}{\sum }}w_{ab}x_{b}+\varphi_{a}\right)+i_{k}$$

The output $u_{k}$ of the kth node of the network output layer:


(14) $$\begin{array}{c} u_{k}=\gamma \left(net_{k}\right)=\gamma \left(\overset{q}{\underset{a=1}{\sum }}w_{ka}y_{a}+i_{k}\right)\\ =\gamma \left(\overset{q}{\underset{a=1}{\sum }}w_{ka}\phi \left(\overset{N}{\underset{b=1}{\sum }}w_{ab}x_{b}+\varphi_{a}\right)+i_{k}\right) \end{array}$$

##### The Back-Propagation Process of the Output Error

The input error propagation process first calculates the output error of each neuron layer from the output stage of the network layer, and then adjusts the weights and thresholds of each neuron layer. According to the step-down method of failure level, the final output of the modified network can be close to the expectation.

The standard function of quadratic error for each sample *p* is $E_{p}$ :


(15) $$E_{p}=\frac{1}{2}\overset{L}{\underset{k=1}{\sum }}{\left(Z_{k}-u_{k}\right)}^{2}$$

The function of the overall systematic error criterion for the training samples of *P* should be:


(16) $$E_{p}=\frac{1}{2}\overset{P}{\underset{p=1}{\sum }}\overset{L}{\underset{k=1}{\sum }}{\left(Z_{k}^{p}-u_{k}^{p}\right)}^{2}$$

According to the error gradient descent method, the correction amount ΔWka of each weight value of the output layer, the correction amount Δak of each threshold value of the output layer, the correction amount ΔWab of each weight value of the hidden layer, and the correction amount $\Delta \varphi_{a}$ of each threshold value of the hidden layer are sequentially adjusted.


(17) $$\Delta w_{ka}=-\mu \frac{ℜE}{ℜw_{ka}};\Delta i_{k}=-\mu \frac{ℜE}{ℜi_{k}};\Delta w_{ab}=-\mu \frac{ℜE}{ℜw_{ab}};\Delta \varphi_{a}=-\mu \frac{ℜE}{ℜ\varphi_{a}}$$

## Experiment of Interior Color and Space Humanized Design Based on Emotional Needs

**Figure 5:**
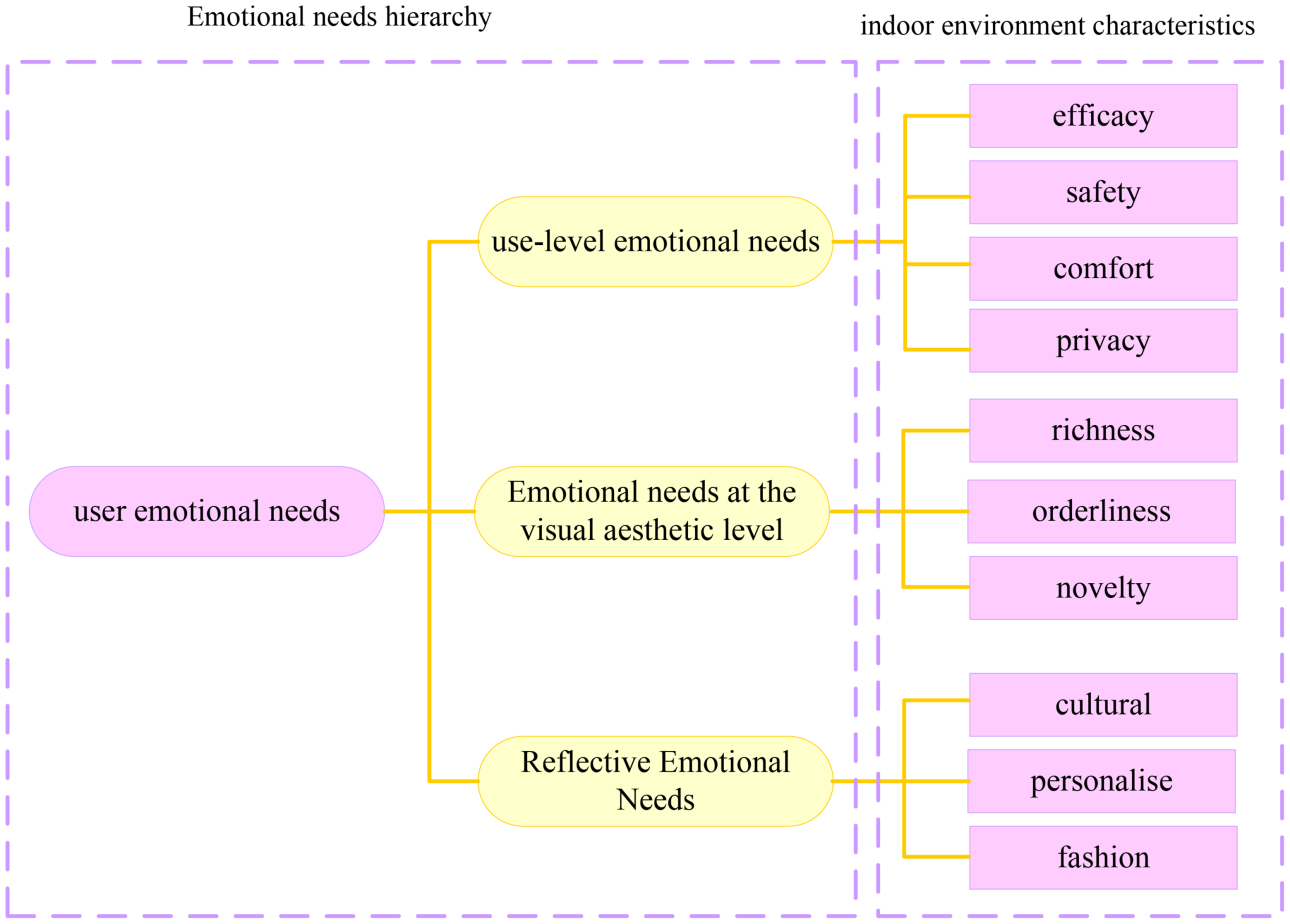
The emotional demand system of indoor environment users.

According to the emotional need level of indoor environment users and the internal environment characteristics related to emotional needs, the emotional need system of indoor environment users is initially constructed, as shown in Figure 5.

### Survey Questionnaire

A total of 198 sample questionnaires were collected in this survey, and multiple-choice responses to the questionnaire data or responses with less serious suspicions were all rejected. Among them, there were 198 valid questionnaires, and the recovery rate was 100%.

The age is divided into three categories: under 24 years old, 24–50 years old, and over 50 years old.

The income level (monthly income) is divided into three categories: below 2,800 yuan, 2,800–5,000 yuan, and above 5,000 yuan.

The educational level is divided into three categories: high school and below, junior college and undergraduate, and undergraduate and above.

The composition of the research samples is presented in Table 1.

**Table 1:** Basic information of the survey samples.

Category	Classification	Number of people (person)	Proportion (%)
Age	Under 24	102	50.51
	24–50 years old	54	27.27
	Over 50 years old	42	21.21
Income level (monthly income)	Below 2,800 yuan	111	56.06
	2,800–5,000 yuan	46	23.23
	More than 5,000 yuan	41	20.71
Education level	High school and below	16	8.08
	College and Undergraduate	108	54.55
	Bachelor degree or above	74	37.38

The collected questionnaires were used for data registration, analysis, and data classification, and SPSS14.0 software was used for operation.

Due to the constraints of research time and research funds, the selection of research samples is limited to a certain extent. The age group of the research objects is mostly young and middle-aged, the education level is mainly undergraduate, and the income level is mainly the group below 2,800 yuan.

The results of the questionnaire are summarized, and the data are processed to analyze the user’s emotional needs. Table 2 reflects the differences in the degree of emotional needs of users at different levels in the internal environment. The order is: emotional needs at the use level > emotional needs at the visual esthetic level > emotional needs at the reflection level.

**Table 2:** Evaluation of emotional needs of indoor environment users.

Emotional needs	Sample size *N*	Minimum	Maximum	Mean
Use level	198	1.00	5.00	4.24
Visual esthetic level	198	1.00	5.00	4.02
Reflective level	198	1.00	5.00	3.96

From the above data, users have the highest emotional needs at the usage level. Users pay more attention to the practical value of the indoor environment, and hope to obtain a pleasant emotional experience by meeting the needs of practical value.

The user’s emotional needs for the three levels are all above 3.00, and users generally have the above three levels of emotional needs. Therefore, it is reasonable to divide the user’s emotional needs into three levels: use, visual esthetics, and reflection (Tynynyka, 2019).

### Emotional Needs

**Figure 6:**
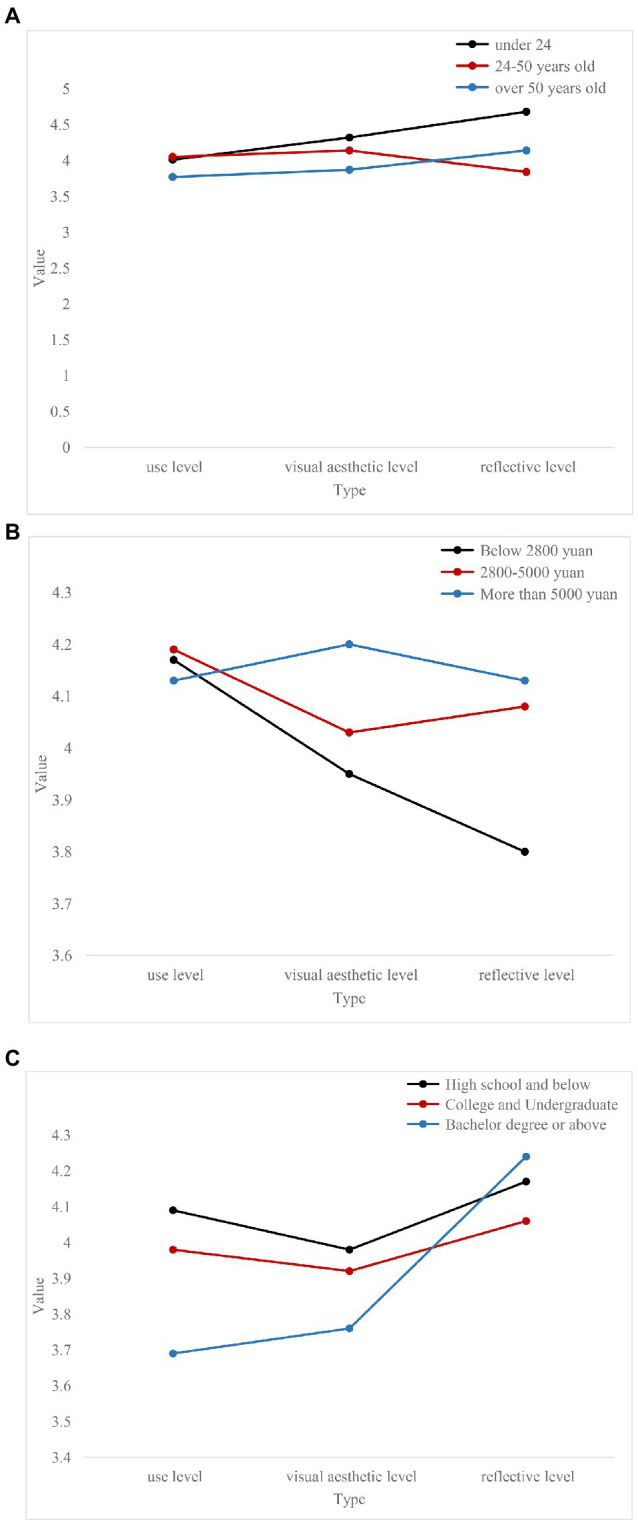
Evaluation of users’ emotional needs from different angles. **(A)** Evaluation of emotional needs of users with different ages. **(B)** Evaluation of the emotional needs of users with different incomes. **(C)** Evaluation of the emotional needs of users with different educational levels.

Next, we will analyze the evaluation of the user’s emotional needs from different angles, as shown in Figure 6.

Figure 6A reflects the correlation between changes in user age and changes in emotional needs. Ascan be seen from Figure 6, as the user’s age increases, the degree of emotional needs increases in both the use level and the reflection level, while the visual esthetic level of emotional needs generally shows a downward trend.

The aging of the user, the hearing loss, the weakening of the sense of smell, the sluggishness of the senses, and the slowness of movement that accompany the physical aging will cause the inconvenience of movement. Older users pay more attention to whether the emotional needs of the indoor environment can be met, and they hope to obtain a good emotional experience in the behavioral interaction in the indoor environment.

The decline in the level of emotional need for visual esthetics in older users is related to visual deterioration caused by aging. With age, changes in the user’s visual organs cause visual impairment. The most significant change in vision is the reduction in the diameter of the cornea, which leads to hyperopia in the elderly. The light transmission ability of the lens of the eye is obviously weakened and the texture becomes hard, which makes the color vision of the elderly deviated to a certain extent, it is difficult to identify similar colors, and the ability to perceive the environment deteriorates. The physiological changes of users limit their access to pleasant visual experience through visual stimuli, resulting in a decrease in the evaluation of emotional needs at the visual esthetic level (Shin et al., 2020).

The age of users and the richness of life experience make them pay more attention to the pleasure at the spiritual level, hoping to obtain more pleasure from thinking in the indoor environment, and their emotional needs at the reflection level are constantly increasing.

Figure 6B reflects the correlation between changes in users’ income levels and the degree of emotional needs. It can be seen from this that there is no obvious correlation between the improvement of the user’s income level and the evaluation of the level of emotional needs at the user level, while the level of emotional needs at the visual esthetic level and reflection level is increasing. For users with a good income level, when the material needs are better satisfied, they should pay more attention to the esthetic value of the indoor environment. Whether the indoor environment can meet own esthetic needs, showing own identity, hobbies, status, and other characteristics is placed in an increasingly important position, and it is hoped that it will have a pleasant feeling in the process of satisfying esthetic needs.

Figure 6C reflects the correlation between users’ educational level and changes in emotional needs. The improvement of the user’s education level is positively correlated with the level of emotional needs at the reflection level, and the users with higher education level pay more attention to the satisfaction of the spiritual needs of the indoor environment. The correlation between users’ educational level and emotional needs at the level of use and visual esthetics is not clear.

**Figure 7:**
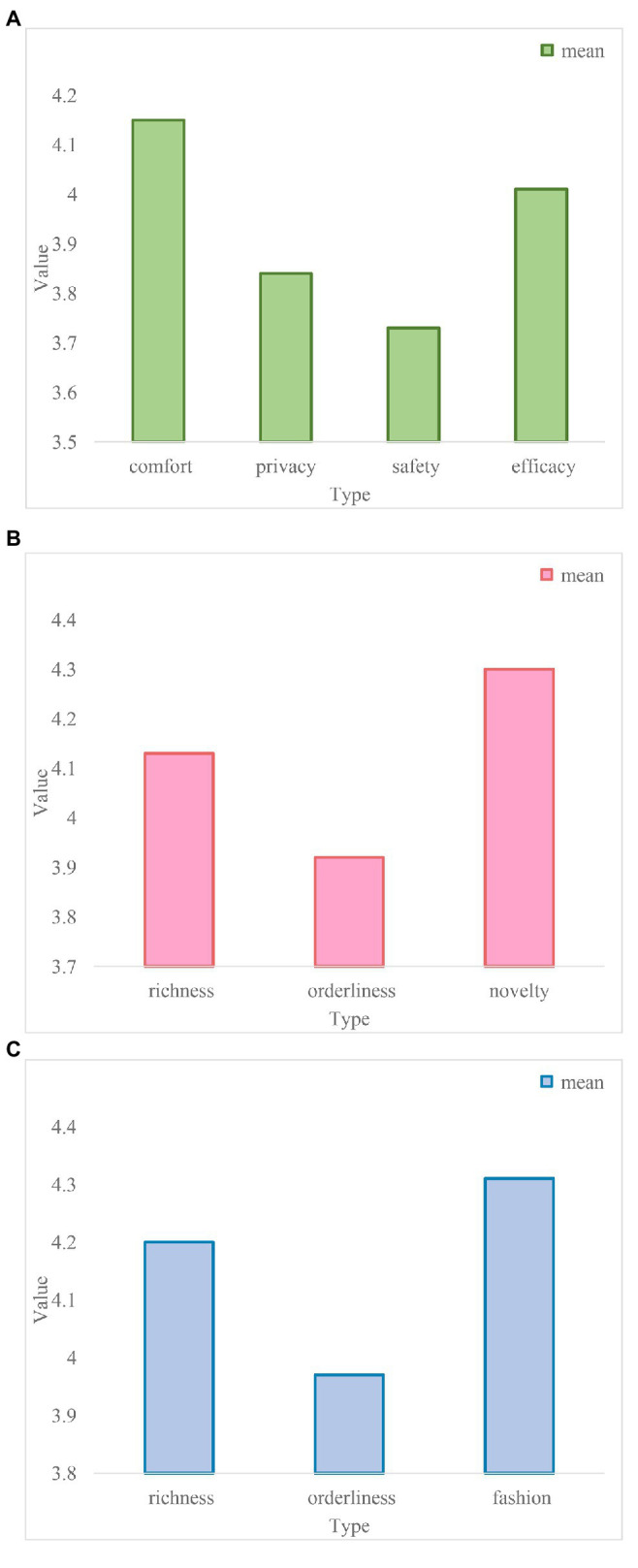
Evaluation of the importance of indoor features at different levels. **(A)** Evaluation of the importance of indoor features at the usage level. **(B)** Evaluation of the importance of indoor features at the level. **(C)** Evaluation of the importance of indoor features at the reflection level.

According to the survey results, Figure 7 shows the user’s evaluation of the importance of indoor features at different levels.

According to the survey results, Figure 7A reflects the user’s evaluation of the role of the indoor features “comfort,” “privacy,” “safety,” and “efficacy” at the usage level in meeting emotional needs. The order is: comfort > efficacy > privacy > security.

The “safety” feature should be the most basic feature of the indoor environment. However, from the results of the user’s evaluation of the importance of the use-level features, the “safety” feature ranks last in the importance of meeting the user’s emotional needs. There should be a neglected situation regarding the role of the user’s “safety” feature in meeting the emotional needs at the user level. Based on this result, the author conducted a return visit to some users who rated the importance of the “safety” feature as low. The results show that this part of the research subjects are generally relatively young, and their good physiological condition makes their activities in the indoor environment without too many dangers and difficulties. And they believe that “safety” should be one of the most basic characteristics of the indoor environment, and no special emphasis is needed. The “safety” feature does not have a strong effect on the emotional stimulation of such users, so they do not have a high evaluation of the importance of the “safety” feature in the indoor environment (Erol and Basar, 2021).

However, designers should not ignore the important role of “safety” in meeting the emotional needs of users when designing. The guarantee of “safety” in the indoor environment is the prerequisite for the realization of the other three features. It is difficult to imagine that users can obtain higher work efficiency, comfort, privacy, and pleasant emotional experience in an environment that feels “unsafe.” The “safety” feature stimulates the user’s sense of security and control, which enables the user to gain the courage and confidence to move and “explore” in the indoor environment.

The “comfort” feature plays the strongest role in meeting the emotional needs at the user level, and the user’s comfort is often achieved through the satisfaction of physiological needs. The materials that come into contact with the user in the indoor environment, the physical environment, and the size of the furniture will affect the user’s physiological state to varying degrees. When designing, designers should take the stimulation of users’ comfortable physiological feelings as a key consideration to meet the emotional needs of users.

Figure 7B reflects the user’s evaluation of the importance of “richness,” “orderliness,” and “novelty” indoor features that meet the emotional needs of visual esthetics, and the order is novelty > richness > orderliness.

Figure 7C reflects the user’s evaluation of the role of “cultural,” “individualized,” and “fashionable” indoor characteristics at the reflective level in meeting the emotional needs of the reflective level, and the order is fashion > cultural > personality.

### Interior Color Design and Space Humanized Design

In interior environment design, when designers choose colors, the first thing to understand is the relationship between color and feel. Reasonable color matching will make people feel positive emotions. Therefore, the first principle of using color in different interior environment design is to meet the needs of users.

The indoor environment is mainly composed of living room, bedroom, kitchen, bathroom, and other spaces. The living room is the main area, which has various functions such as leisure, entertainment, meeting guests, and activities. Therefore, designers can mainly use warm colors according to people’s common emotional principles in color, so as to provide users with warm and harmonious emotional support and create a warm atmosphere. At the same time, it is necessary to reasonably distribute the proportion of main colors and monochrome in the whole environment space. In general, when using colors such as yellow, orange, red, etc., as the main color, the color of the sheet should choose green and similar low-light colors to create a warm environment. In daily life, warm colors in the living room are more common. Bright colors not only give a warm feeling, but also reflect the hospitality of the host. According to user needs, it can be roughly divided into hot (red as the main color, green and purple as the auxiliary colors), cheerful (yellow and blue as the main colors, green and warm gray as the auxiliary colors), comfort (blue is the main color, warm gray is the secondary color), dynamic (orange and red are the main colors, yellow is the bed linen color), and other main types of living room colors.

The main function of the bedroom is to rest, and the choice of color also varies by age. For the elderly, blue-gray, brown, white, and other colors are generally recommended. Since choosing a mature and stable color can easily make users feel quiet and comfortable, for young people, it is generally recommended to use warm colors, such as yellow, pink, white, etc. Because the choice of bright colors is conducive to conveying a warm and romantic feeling, the color selection of children’s rooms should usually be close to the innocent character of children, and use more colors and higher color brightness. Designers should also choose colors reasonably based on factors such as users’ personal needs and cultural heritage (Lan et al., 2019).

As a frequently used functional space, the kitchen should be matched with colors to facilitate the user’s meal. The main color is yellow, orange, and other stimulating colors, which is conducive to allowing users to create delicious stimulation through contact and feel a warm dining environment. Considering the practicality, since the kitchen is an environment with a fire source, in the color matching process, it should be considered that it affects the user’s feeling through color, and use medium and high brightness colors for logical color matching.

As a more private setting than the bedroom—the bathroom, which is usually smaller and mostly cool toned. Using brighter colors in smaller spaces can produce wider views and brighter effects. In terms of design, designers can use the textures of different materials to improve the feeling of uniform tones, provide a safe and comfortable user environment, and at the same time meet the privacy and emotional needs of users.

Most of the time, people have high emotional needs for the security, privacy, and sense of domain of their living environment. The physical security of most groups is closely related to the security of their living indoor environment, while the psychological security is directly related to the privacy and sense of domain of the indoor environment. To improve the physical security of the occupants, it is necessary to do a good job in safety design; to improve the psychological security of the occupants, it is necessary to enhance the privacy and the occupant’s sense of domain through the reasonable separation of the indoor space.

Generally speaking, women spend more time at home than men. If want to make the occupant’s life no longer lonely and boring, it is necessary to bring more functional enjoyment to the occupant in the limited indoor space, so as to enrich the life and reduce the loneliness in the process of living alone. Therefore, the functional design of the interior should be more diversified. In the limited indoor space, it is particularly important to expand the space and enhance the efficacy.

In the indoor environment, comfort is simply a feeling that is beneficial to the survival function of the human body formed after the human body is stretched and relaxed. It requires that the indoor space environment should comply with the principles of convenience and pleasantness in use, reasonable and effective operation, safe and barrier-free behavior, and scientific visual response. According to the needs of the occupants’ physical characteristics and living habits, the space form and the shape, color, and scale of the furniture and equipment should be determined to meet the occupants’ flexible and stretched use requirements. Scientific and reasonable arrangement and installation of various furniture facilities and sanitary ware, kitchen utensils, and other household equipment can achieve the best use effect and operation function (Zhang et al., 2020). At the same time, thermal factors, glare, noise, and air freshness in the indoor environment also have a great impact on human comfort. Therefore, scientific control of the indoor physical environment is also particularly important for the improvement of indoor comfort.

With the rapid development of science and technology, the application of smart home makes up for indoor shortcomings and deficiencies in living life from different angles and in many ways. It improves the comfort, convenience, and safety of the indoor environment, enabling occupants to obtain the most intelligent living experience and feel relaxed, convenient, and pleasant in their living life.

## Discussion

Through the study of emotional design literature works, this paper has initially mastered the relevant basic knowledge, and analyzed how to research the color factors and space humanization in interior design based on emotional needs. The concept and algorithm of artificial neural networks are expounded, the learning method is studied, the BP neural network is explored, and the applicability of emotional needs in interior design is analyzed through experiments.

Designers must consider many factors when designing a space, such as space layout, material selection, technology, engineering cost, practical value, etc. However, many designers are more concerned with the functioning of the space than the emotional appeal. The success of space design depends not only on the convenience of the space, but also on the importance of the design, which only has emotional and spiritual resonance to maximize the design spirit. Therefore, the emotional factor in the design is more important than the practical factor.

Through the experimental analysis, this paper shows that through the classification of the survey data, the user’s evaluation value of the importance of the internal environment features to meet the emotional needs is above 3.00 and about 4.00, respectively. Users believe that reported indoor environmental characteristics will play a more important role in satisfying emotional needs.

## Conclusion

The purpose of design is to meet the physical and psychological needs of users and must be the driving force behind human design. Emotion is an important factor that constitutes the human psychological cognitive system. Interior decoration-themed spaces that adapt to emotional needs should make full use of psychology, environmental behavior, and innovative design techniques to create spaces. It should pay attention to the choice of building and interior decoration materials, color matching and humanized design of space, the perception of the environment through the senses, and the planning of emotional care. This work attempts to analyze emotional needs and the current state of interior space design, and refer to the psychological characteristics of people’s behavior in order to trigger emotional needs and design thinking for themed interior spaces. Let people’s needs not only stay on the level of material needs, but also pay more attention to spiritual needs, so that people can relax, maintain, imagine, and perceive the interior, the theme of interior design has been further developed, and promoted the development and rise of emerging industries.

## Data Availability Statement

The original contributions presented in the study are included in the article/supplementary material; further inquiries can be directed to the corresponding author.

## Author Contributions

SW guided the research directions and ideas. YX writing, static analysis of data, and experimental operation. All authors contributed to the article and approved the submitted version.

## Funding

This study was supported by Guangdong Quality Engineering and Teaching Research and Teaching Reform Project “Environmental Design Characteristic Major” of (CXQX-ZI201802), a key cultivation and construction discipline in Guangdong Province the project “Design Art” (Guangdong Education Research Letter 2012.13), and the key scientific research platform project of “Green Environment Art Design Research Center” (2440314) of Guangdong Baiyun University.

## Conflict of Interest

The authors declare that the research was conducted in the absence of any commercial or financial relationships that could be construed as a potential conflict of interest.

## Publisher’s Note

All claims expressed in this article are solely those of the authors and do not necessarily represent those of their affiliated organizations, or those of the publisher, the editors and the reviewers. Any product that may be evaluated in this article, or claim that may be made by its manufacturer, is not guaranteed or endorsed by the publisher.
